# Formation of Fe-Ni Nanoparticle Strands in Macroscopic Polymer Composites: Experiment and Simulation

**DOI:** 10.3390/nano11082095

**Published:** 2021-08-18

**Authors:** Ruksan Nadarajah, Leyla Tasdemir, Christian Thiel, Soma Salamon, Anna S. Semisalova, Heiko Wende, Michael Farle, Stephan Barcikowski, Daniel Erni, Bilal Gökce

**Affiliations:** 1 Technical Chemistry I, Center for Nanointegration Duisburg-Essen (CENIDE), University of Duisburg-Essen, Universitaetsstr. 7, 45141 Essen, Germany; ruksan.nadarajah@uni-due.de (R.N.); cakir_leyla@hotmail.com (L.T.); stephan.barcikowski@uni-due.de (S.B.); 2 General and Theoretical Electrical Engineering (ATE), Center for Nanointegration Duisburg-Essen (CENIDE), University Duisburg-Essen, 47048 Duisburg, Germany; christian.thiel.ate@uni-due.de (C.T.); daniel.erni@uni-due.de (D.E.); 3 Faculty of Physics, Center for Nanointegration Duisburg-Essen (CENIDE), University of Duisburg-Essen, Lotharstr. 1, 47057 Duisburg, Germany; soma.salamon@uni-due.de (S.S.); anna.semisalova@uni-due.de (A.S.S.); heiko.wende@uni-due.de (H.W.); michael.farle@uni-due.de (M.F.); 4 Materials Science and Additive Manufacturing, University of Wuppertal, Gaußstr. 20, 42119 Wuppertal, Germany

**Keywords:** laser ablation, FeNi, strand, COMSOL, polymer composite

## Abstract

Magnetic-field-induced strand formation of ferromagnetic Fe-Ni nanoparticles in a PMMA-matrix is correlated with the intrinsic material parameters, such as magnetization, particle size, composition, and extrinsic parameters, including magnetic field strength and viscosity. Since various factors can influence strand formation, understanding the composite fabrication process that maintains the strand lengths of Fe-Ni in the generated structures is a fundamental step in predicting the resulting structures. Hence, the critical dimensions of the strands (length, width, spacing, and aspect ratio) are investigated in the experiments and simulated via different intrinsic and extrinsic parameters. Optimal parameters were found by optical microscopy measurements and finite-element simulations using COMSOL for strand formation of Fe_50_Ni_50_ nanoparticles. The anisotropic behavior of the aligned strands was successfully characterized through magnetometry measurements. Compared to the unaligned samples, the magnetically aligned strands exhibit enhanced conductivity, increasing the current by a factor of 1000.

## 1. Introduction

High purity iron alloy nanoparticles have potential applications in different research domains due to their properties related to electromagnetic shielding [1,2], magnetism [3,4], and catalysis [5,6]. A wide variety of synthesis methods have been developed for the preparation of FeNi nanoparticles. The most popular among these synthesis methods are based on hydrothermal processes [7,8], wet chemistry [9,10], hydrogen plasma reaction [11,12], and redox-transmetalation processes [13]. These synthesis methods are often multi-step, time-consuming processes that lead to nanoparticles that contain residues or ligands that are undesirable for applications in fields such as biomedicine or sensor technology. Laser ablation in liquid (LAL) has been established as an alternative synthesis method, which allows the synthesis of surfactant-free nanoparticles. Furthermore, LAL [14,15,16,17] is a scalable [18,19] and versatile green method capable of producing alloy nanoparticles that have been used as microwave absorbing materials [20], magnetic recording media [21], and transparent and electrical conducting coatings [22]. By introducing these nanoparticles into a polymer matrix [23,24,25], the nanoparticle properties can be combined with the properties of the polymer, opening up innovative possibilities for scientific and industrial applications [26,27,28,29]. The nanoparticle polymer composite is typically produced from a liquid phase in which the Brownian motion governs the motion of superparamagnetic nanoparticles. Furthermore, the magnetic moment of the nanoparticles can be aligned to form nanochains or nanostrands by applying an external magnetic field [30,31,32].

Self-assembly has attracted considerable attention from a growing number of researchers to design functional nanostructures. Several methods have been developed to fabricate nanostructured magnetic materials, including dipole-driven assembly [33,34], magnetic field-induced (MFI) [35,36,37], and template synthesis [38,39]. Of the mentioned methods, MFI assembly is a relatively simple and inexpensive technique for fabricating magnetic nanostrands. If colloidal metallic nanoparticles experience a magnetic field, the interaction of the particles with the external magnetic field (F_m_) tends to align the magnetic dipoles (particles) along the magnetic field direction (parallel to each other). Particle aggregation can be considered as the result of the competition between the Brownian motions of the particles and the dipolar interaction between the particles [32,40]. In contrast, strand formation is considered the outcome of the competition between the magnetic energy, the surface energy, and the entropic contribution of the aggregated chains [41]. Since the potential energy for a particle is much lower near the end of a chain, the particles tend to cluster at the end of the chains; in addition, the attractive force is particularly strong so that the chains are connected and act as two head-to-tail dipoles. Liang et al. suggested that the strands are formed because this is the most energetically favorable particle arrangement compared to a hexagonal lattice arrangement (disordered arrangement) [42,43].

Nanostrands of spherical nanoparticles exhibit distinct magnetic properties due to geometric confinement, magnetostatic interactions, and nanoscale domain formation. The formation of nanostrands from nanoparticles has been proposed as an alternative way of controlling their macroscopic magnetic behavior as needed [30,44,45]. Such chains directly influence effective anisotropy [46], susceptibility [47], and hysteresis losses [45]. These fundamental properties have potential applications in data storage devices, logic devices, and magnetic field sensing. Such systems also provide valuable insight into fundamental physical phenomena and properties of nanoscale magnetism. Reports on the synthesis of FeNi nanoparticles and strand formation of magnetic nanoparticles can be found in the literature [3,30,42]. The strand formation was also demonstrated for further material systems, such as Fe_3_O_4_ [48], Co_3_C [43], Ni [49,50], FePt [42], FeCo [42], FeAu [51], and FeRh [52]. However, to the best of our knowledge, a detailed study on the formation of strands using laser-generated size-controlled nanoparticles in a polymer matrix and predicting the formation by simulations is yet to be performed.

Strand formation within a polymer matrix can be optimized by varying physical properties and material parameters, such as viscosity, magnetic field strength, and nanoparticle size and magnetic moment. Simulations can be used to predict the influence of these material parameters on the behavior of nanoparticles in the polymer matrix. The finite element method (FEM) is a widely used numerical technique for solving engineering and physics problems involving behaviors that can be described by differential equations. These differential equations can also describe a variety of physical phenomena of nanosystems, ranging from electrical [53,54,55] and mechanical systems [56,57,58,59] to thermo [60,61,62] and rheological [63,64,65] problems. Various simulation studies for strand formation exist, e.g., Monte Carlo [66,67], Brownian dynamic [68], or molecular dynamic simulation [69]. However, none of these studies predict the size and length of strand formation depending on material and external parameters. In this study, COMSOL Multiphysics^®^ is used to predict and confirm the effect of different parameters on the formation of FeNi alloy nanostrands in a PMMA-acetone-solution. The influence of different factors, such as the particle size, viscosity, magnetization, and magnetic field strength, on the strand formation, are investigated and compared to experimental results.

## 2. Materials and Methods

**Experiments.** The FeNi nanoparticle colloids were synthesized by LAL. A Fe_50_Ni_50_ foil (Sekels GmbH, Ober-Mörlen, Germany) was immersed in acetone, and LAL was performed with a picosecond pulsed Nd-YAG laser (Ekspla, Vilnius, Lithuania, Atlantic Series, 10 ps, 100 kHz, 80 µJ, 1064 nm). The laser beam was directed into a laser scanner and focused through an f-theta lens (focal length 100.1 mm) onto the FeNi target. The laser beam has a Gaussian profile with an incident laser fluence of 3.5 J/cm^2^. A scanning speed of 6 m/s was chosen to bypass the laser-induced cavitation bubbles spatially. All the experiments were carried out using a 100 mL batch chamber and an ablation time of 15 min. The ablated mass was determined gravimetrically by weighing the target before and after ablation using a microbalance (Precisa, Dietikon, Switzerland, XT 220 A). Since the synthesized FeNi particles have a broad size distribution, they were separated according to their size by stepwise centrifugation, using a Hettich Zentrifugen Universal 32 R centrifuge (Table 1). The colloids were then brought to a final concentration of 0.4 g/L. Their composition was subsequently verified by X-ray fluorescence measurements.

To produce FeNi nanostrand-polymer composites, a 5 wt% poly(methylmethacrylate) (PMMA) with FeNi nanoparticles in acetone was prepared. A nanoparticle concentration of 0.2 wt% in acetone was used. The PMMA-acetone solution containing FeNi nanoparticles was then dried on a glass substrate under an external magnetic field with a flux density of 170 mT. After drying, the formed nanostrands were imaged using an optical microscope (CX 40, Olympus, Shinjuku, Japan). The experimental validation involved measuring 500 strands per test point. The magnetic properties of the FeNi nanoparticles were investigated using a Quantum Design SQUID-MPMS magnetometer (Quantum Design GmbH, Darmstadt, Germany), and the composite materials were investigated by the vibrating sample magnetometer (VSM) option of a Quantum Design PPMS DynaCool, using the large bore option to fit an app. 10 mm × 10 mm piece of composite material.

**Figure 1:**
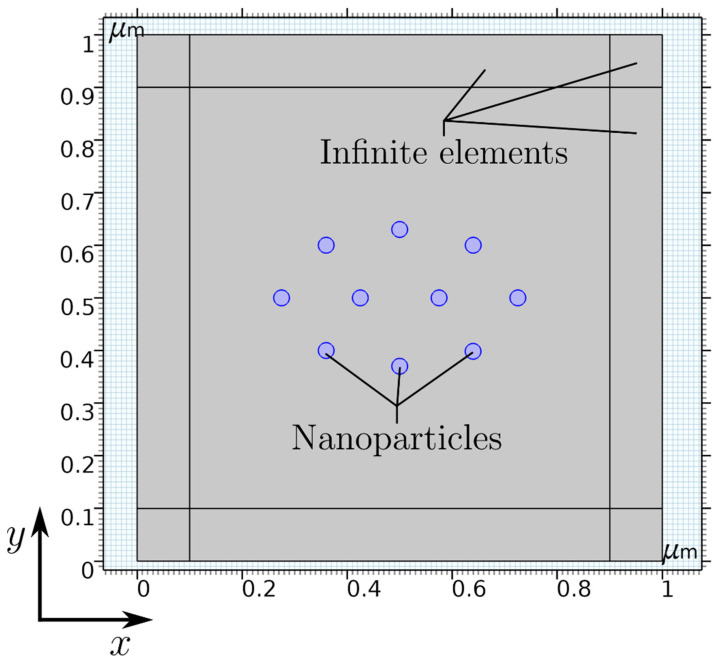
Simulation setup of 10 symmetrically distributed nanoparticles in a 2D computational domain with infinite elements in COMSOL Multiphysics.

**Simulation.** The simulation was carried out using the finite-element method (FEM) software COMSOL Multiphysics, in which the “magnetic fields, no currents” (mfnc) physics within the AC/DC module is chosen to model the inter-particle interaction in the presence of a permanent magnet. The simulation setup is presented in Figure 1, consisting of 10 symmetrically distributed nanoparticles (blue circles) with a radius of r_p_ = 15 nm each in a 2D computational domain. To implement the influence of the particle concentration, a distance between two particles of 104 nm is defined (Figure S1 in the Supplementary Materials). Additionally, a layer with a thickness of 100 nm at each boundary of the simulation setup is utilized using the infinite element boundary condition to account for an approximately infinitely large computational domain.

Furthermore, it should be noted due to the 2D simulation setup, nanocylinders with a user-defined out-of-plane thickness of 15 nm are calculated instead of spherical nanoparticles to reduce the numerical demand/effort. However, in the center-plane of spherical nanoparticles, the magnetic field solution provides similar results to the cylindrical nanoparticles in the 2D simulation. Hence, it can be used as a valid approximation for the particle shape.

This 2D continuum model uses the following equation for a static magnetic field solution in the time domain for the magnetic scalar potential *ψ*_m_ and the magnetic permeability *μ*:

(1) $$\nabla \cdot \left(\mu \nabla \psi_{m}\right)=0$$

The magnetic field generated by the permanent magnets is defined in COMSOL by a homogenous background magnetic flux density B_b_, set to a value of B_b_ = 170 mT, which matches experimental measurements at the centerline between the two permanent magnets.

To accurately model the occurring particle movements, multiple forces need to be taken into account, which can be summarized to a total force formulation shown in Equation (2):

(2) $$F_{\mathrm{total}}=F_{D}+F_{B}+F_{\mathrm{Mag}}$$

here, *F*_D_ represents the drag forces acting on each particle in a fluid with a specific diameter *d*_p_ and can be calculated as:

(3) $$F_{D}=\left(\frac{1}{\tau_{p}}\right)m_{p}\left(u-v\right)$$

(4) $$\tau_{p}=\frac{\rho_{p}d_{p}^{2}}{18\mu }$$

with *u* − *v* describing the acceleration of the particle, μ being the viscosity of the fluid, and *m*_p_ and $\rho_{p}$ illustrating the mass and density of the particle. The Brownian force *F*_B_ can be calculated using:

(5) $$F_{B}=\zeta \sqrt{\frac{12\pi k_{B}\eta Tr_{p}}{\Delta t}}$$

including the Boltzmann constant kB and the temperature *T*. Since the Brownian movement is random and undirected, a random seed ζ, depending on the timestep Δ*t*, for each particle and coordinate was implemented additionally. Finally, the magnetic force *F*_Mag_ is evaluated using Equation (6):

(6) $$F_{\mathrm{Mag}}=\underset{\partial \Omega }{\int }nT_{S}dS\;$$

This force uses Maxwells’ stress tensor *T*_S_ in combination with the surface normal $\vec{n}$ to formulate the projected stress tensor $\vec{n}T_{S}$ on the desired surface *S*. The projected stress tensor is calculated in COMSOL as:

(7) $$nT_{S}=-0.5n\left(H\cdot B\right)+\left(n\cdot H\right)B^{T}$$

with ***H*** being the magnetic field and *B* the magnetic flux density, respectively. Integrating this tensor over the boundary of the domain Ω leads to the desired magnetic force at the particle surface ***S***.

The resulting movement of the particles experiencing said forces is realized by implementing a combination of a hyperelastic moving mesh and an automatic remeshing node, which enables a free movement of each particle through the deformation of the mesh. If the mesh quality is below a certain threshold, resulting in poor simulation results or converging problems, the mesh is automatically rebuilt to prevent said issues. The necessary particle velocity $v_{p}$ is then calculated using the mesh displacement at each particle given by COMSOL in combination with an ordinary differential equation (ODE) formulation to solve for the particle velocity using Newton’s second law:

(8) $$F_{\mathrm{total}}=m_{p}\frac{d}{dt}v_{p}$$

in combination with Equation (2) now resulting in:

(9) $$m_{p}\frac{d}{dt}v_{p}-F_{D}-F_{B}-F_{\mathrm{Mag}}=0$$

now being able to calculate the particle velocity $v_{p}$.

**Figure 2:**
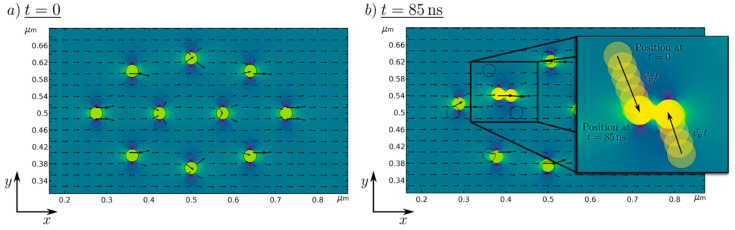
Numerically simulated magnetic scalar potential of 10 Fe_50_Ni_50_ nanoparticles in a magnetic field at (**a**) *t* = 0 s, and (**b**) *t* = 85 ns. The black arrows represent the magnetic flux density.

To visualize the working numerical particle movement and strand building, the magnetic field solution and particle position for two different time steps are shown in Figure 2.

At *t* = 0 (left) the starting position of the nanoparticle in the presence of the magnetic background field is presented, where no resulting movement of the particle takes place. For an increasing time, the nanoparticles move towards another, which is shown at an exemplary time at *t* = 85 ns. It can be seen that particles are moving in the direction of neighboring nanoparticles and are getting close enough to allow strand formation. This, proves the general plausibility of the simulation setup and helps to visualize and further understand the strand formation process.

## 3. Results

### 3.1. Effect of Particle Size Distribution

**Figure 3:**
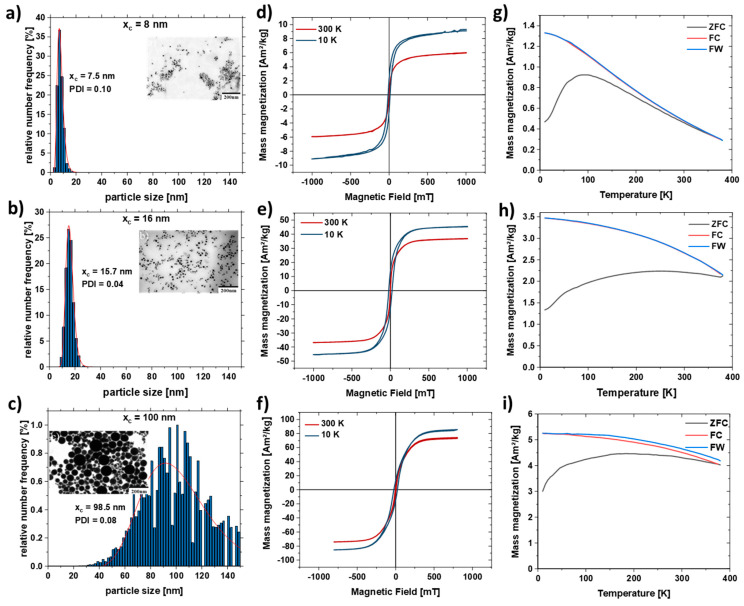
SEM images and particle size distributions of Fe_50_Ni_50_ nanoparticles obtained after centrifugation of the as-is nanoparticle colloid with different RPMs to separate different size fractions with an average diameter of (**a**) 7.5 nm, (**b**) 15.7 nm, (**c**) 96.5 nm and their respective M(H) curve (**d**–**f**) at 300 K (red) and 10K (blue). (**g**–**i**) Temperature-dependent field cooled (FC, red), zero field cooled (ZFC, black) and field warmed (FW, blue) magnetization curves measured in 2 mT field for the respective FeNi nanoparticles.

Laser ablation in liquids is a scalable method and a versatile process to produce FeNi nanoparticles [42,70]. Laser-generated Fe_50_Ni_50_ nanoparticles typically have a large particle size distribution (Figure S2). As expected for LAL, the mass-weighted particle size distribution shows a bimodal distribution due to the different ablation mechanisms [71] with peaks at 15 nm and 60 nm. To investigate the influence of the particle size distribution on the strand formation, the particles were size separated by centrifugation (Figure 3). Three different particle distributions were separated using the centrifugation protocol described in Table 1. As shown in the scanning electron microscopy (SEM) images in Figure 3, the centrifugation process resulted in three different size distributions with mean diameters of 7.5 ± 2.4 nm, 15.7 ± 3.1 nm, and 98.5 ± 28.1 nm. All colloids show a polydispersity index (PDI) smaller than 0.3 after separation; thus, all particle size distributions can be considered monomodal. In addition, EDX analysis to characterize the FeNi nanoparticles was performed (Figure S3). The line profiles of the composition and the mapping of Fe and Ni on a Fe_50_Ni_50_ nanoparticle show that the FeNi alloy has the aimed equimolar composition. More information on the LAL-synthesized Fe_50_Ni_50_ nanoparticles can be found elsewhere [72].

Figure 3d–f shows the hysteresis loops M(H) measured at 300 and 10 K for the size-separated samples (Table 1). The high field magnetization increases with the size of FeNi particles, at room temperature reaching 6, 37, and 74 Am^2^/kg for x_c_ = 8 nm, x_c_ = 16 nm, and x_c_ = 100 nm, respectively, ensuring a stronger stray field for larger particles. The increase of high field magnetization at low temperatures typical for ferromagnetic nanoparticles is observed for all three measured ensembles and most pronounced for the particles with the smallest diameter. A superparamagnetic behavior was observed at room temperature for the smallest nanoparticles (x_c_ = 8 nm), as evidenced by the negligible coercivity (Figure 3d) and the shape of Zero-Field-Cooled and Field-Cooled (ZFC-FC) magnetization curve (Figure 3g), reveals a broad maximum at temperatures below 100 K. The open hysteresis M(H) loop at 10 K confirms the “blocked” ferromagnetic state. ZFC-FC curves for larger particles (Figure 3h,i) reveal a very broad, plateau-like maximum within 10–390 K, which along with open hysteresis M(H) loops at 300 and 10 K, reflects their ferromagnetic state (no superparamagnetism). Figure S4 shows the hysteresis curve of the non-centrifuged sample, where it has a high field magnetization of 52 Am²/kg due to the bimodal particle size distribution.

**Figure 4:**
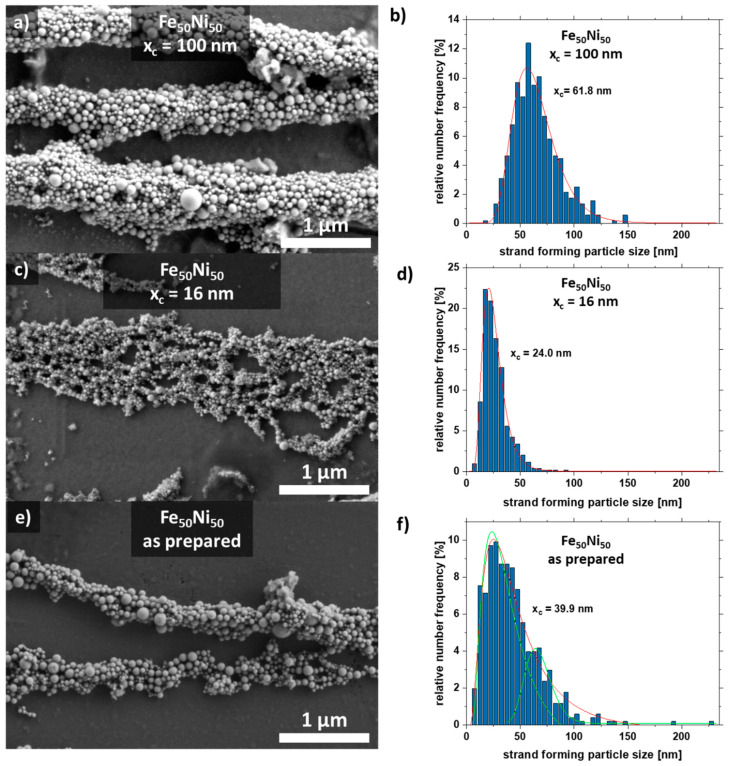
SEM images of strands and their respective particle size distribution extracted from the strands of the SEM formed by Fe_50_Ni_50_ colloids with particle sizes of (**a**,**b**) x_c_ = 100 nm, (**c**,**d**) x_c_ = 16 nm, and (**e**,**f**) as-prepared.

Scanning electron microscopy images were taken to analyze the aspect ratio and particle size distribution of the nanoparticles forming the strand (Figure 4). For this purpose, the strands were formed on a glass substrate without PMMA. The strands with the large particles (x_c_ = 100 nm) form well-formed cylindrical strands due to the high magnetic attraction (Figure 4a), while the colloid with a mean particle size of x_c_ = 16 nm forms a network-shaped strand (Figure 4c). As a comparison, the strand from the as-prepared colloid is shown in Figure 4e, which has features of both Figure 4a,c. It also forms cylindrical strands that are connected in a network.

Furthermore, the particle size distribution in the strand was analyzed (Figure 4). The colloid with the large particles x_c_ = 100 nm forms strands in which particles with a diameter of ~60 nm dominate. The colloid with the medium-sized particles x_c_ = 16 nm forms strands mainly with particles of size 24 nm. Interestingly, there is a large fraction (4.8%) of large particles (50–60 nm). The raw colloid forms strands with the majority of particles having a diameter of x_c_ = 39.9 nm. However, there are also many particles in the size range of 50–60 nm. This indicates that particles in the size range of 20–25 nm and 50–60 nm make a large contribution to strand formation, which is also visible for the raw colloid (Figure 4f). Strand formation was also investigated with small particles (x_c_ = 8 nm), although no strands were formed here due to the paramagnetic character of the particles (Figure S5).

**Figure 5:**
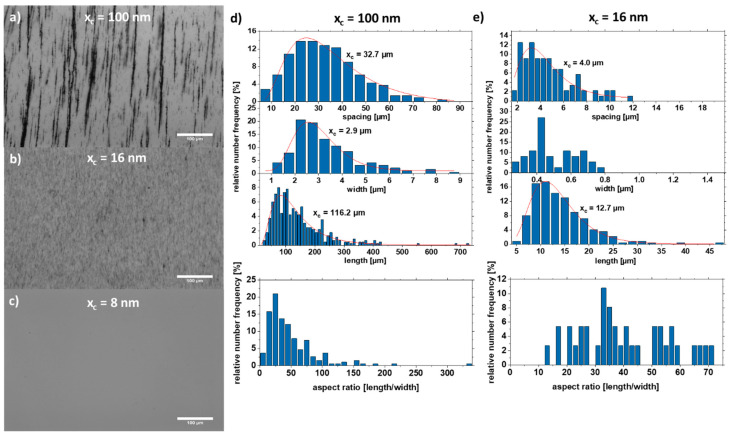
Optical microscopy images of strands in 5% PMMA solution with particle sizes (**a**) x_c_ = 100 nm, (**b**) x_c_ = 16 nm, and (**c**) x_c_ = 8 nm. The length, width, spacing, and the aspect ratio of the respective strands are shown for (**d**) x_c_ = 100 nm and (**e**) x_c_ = 16 nm.

The motion is mainly described by the random Brownian molecular motion, and agglomerates form during drying without an external magnetic field. When an external magnetic field is applied, the particles are magnetized, and the additional dipole–dipole interaction causes the particles to form strands. Strand formation is preferred for larger particles but critical for small particles due to their weak magnetic interaction. In Figure 5, the strand length, width, and aspect ratio of strands formed by the particles shown in Figure 4a (large particles, x_c_ = 100 nm) and Figure 4c (medium-sized particles, x_c_ = 16 nm) were extracted by analysis of optical microscopy images (Figure 5a–c). The large particles form very long strands (with an x_c_ value of 116.2 µm) with strands larger than 700 µm and a width of ~2.9 µm. However, the medium-sized particles form smaller strands (x_c_ = 12.7 µm) with a width of ~0.5 µm. The strand growth can be described by a connection and coarsening model [32]. Due to their smaller size, the magnetic attraction of the particles is lower, and the formed strands cannot “connect” quickly enough to increase the strand length. As a result, the distance between the strands is also smaller by a factor of eight because the strands have not yet been connected, whereas the attraction of the large particles is so strong that the strands connect as soon as they come close. A second possible reason is that the attraction regime of the small particles is smaller, and the strands can therefore come closer without being in the area of attraction. Figure 2 shows the position of the particles at two different time intervals (t = 0 s, and t = 85 ns). In the simulation, the particles move randomly until they come within their attraction radius (Figure 2b), where the ordering attraction force is stronger than the disordering Brownian force, resulting in strand formation. In Figure 5c, particles smaller than 10 nm were used to demonstrate that no strands are formed. This can be explained on the one hand by the superparamagnetic state of the particles (Figure 3f). On the other hand, the smaller size of the particles results in a smaller radius of attraction. As a result, the particles do not “see” the other particles magnetically, and thus no strand formation occurs.

**Figure 6:**
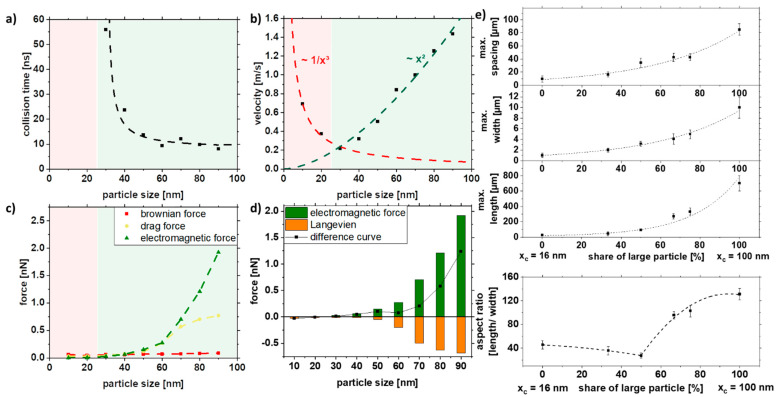
COMSOL simulation data of (**a**) collision time, (**b**) velocity, and (**c**,**d**) acting forces in dependence of the particle size and experimental validation of (**e**) length, width, spacing, and aspect ratio of strands as a function of particle size distribution in a 5% PMMA solution.

Figure 6 shows the simulation results depending on the particle size. The duration until the first collision, the velocity, and the forces (Brownian, drag, and electromagnetic force) were analyzed. The collision time is a qualitative indicator of whether a strand is formed or not. The red area represents the areas where no collision could be simulated in the given simulation time and thus not resulting in strand formation. As seen in Figure 6a, below 20 nm, no collision could be observed, which was also confirmed experimentally (Figure 5). In addition to the collision time, the velocity of the particles will give further insight into particle movement. It can be seen that the velocity of the particles increases quadratically after a particle size of 30 nm (Figure 6b). For particles with sizes less than 30 nm, the speed increases with decreasing particle size. This can be explained by the fact that the electromagnetic attraction increases with the radius r² and is stronger for large particle sizes. At the same time, the competing Brownian motion decreases with 1/r³ and is stronger for small particle sizes. Since these competing mechanisms are most effective in different size ranges, it results in a minimum at 30 nm. To predict the strand formation, the electromagnetic force was equated with Brownian motion in the Langevin equation. The fundamental equation to describe the interaction of the Brownian particles with their environment is called the Langevin equation, which contains both frictional and random forces. A simplified Langevin equation was used, consisting of the Brownian force *F*_B_ and the drag force *F*_D_.

(10) ***F***_L_ = ***F***_B_ + ***F***_D_

By plotting the difference curve between the ordering electromagnetic force and the opposing Langevin force *F*_L_, we can obtain information about the strand formation.

Positive values for the difference curve indicate strand formation, while negative values indicate that strands are not being formed due to the predominant Brownian motion. Furthermore, the increase in magnitude of the difference curve for larger particle sizes indicates the formation of longer strands.

The simulation can be experimentally verified, as shown in Figure 6e. Note that the simulations were performed only with one particle size due to simplicity, as a proper distribution of particles would increase the simulation space, thereby increasing simulation length and resources, while the colloids used in the experiments have a specific particle size distribution that also affects strand formation (Figure 3). Furthermore, for the control of length, width, and spacing, the colloid was mixed with the large particles (x_c_ = 100 nm) and the medium-sized particles (x_c_ = 16 nm), which reflects the increasing particle size in the simulations (Figure 6e). It can be seen that the length, width, and spacing of the strands can be controlled with the particle size distribution. More prolonged and broader strands are formed with increasing particle size. However, the spacing of the particles also increases along with the size distribution of the strands. By mixing large and medium-sized particles, relatively long strands with small distances can be realized, as indicated in Figure 4e. Due to the network formation, the strands are closer to each other, which can be particularly interesting for electrical conductivity.

In summary, the simulation cannot predict the actual strand length but can describe the quality of the influencing factor. It is evident that the difference curve (Figure 6d) goes below 20 nm in the negative range so that no strand formation is possible, and above 20 nm, the strand length increases with x^2^. This could be validated in the experiment.

### 3.2. Effect of Polymer Viscosity during Formation

**Figure 7:**
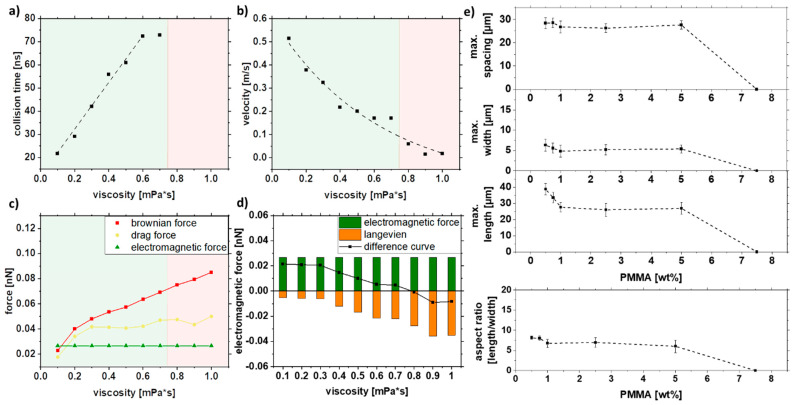
COMSOL simulation data of (**a**) collision time, (**b**) velocity, and (**c**,**d**) forces in dependence on the viscosity and experimental validation of (**e**) length, width, spacing, and aspect ratio of strands as a function of the PMMA concentration for a mean particle size of x_c_ = 16 nm.

As particle motion depends on the viscosity of the liquid, different viscosities were set up in the simulation (Figure 7a–d). Since no strand formation could be simulated for particles smaller than 30 nm in Figure 6 and there is a relatively small proportion of 8% of the particles is larger than 20 nm in the colloid x_c_ = 16 nm, the following simulation was performed with a constant particle size of 30 nm. The Brownian motion decreases with 1/η (Figure 7b); for 0.8 mPa*s and higher viscosities, no collision was observed (Figure 7a). As the increase of viscosity does not affect the electromagnetic force, only the Brownian and drag forces are increasing with higher viscosity (Figure 7c). Interestingly, for viscosities below 0.3 mPa, no change in the difference curve (Figure 7d) was observed, indicating no change in the strand length.

As the PMMA concentration can influence the viscosity in the solution, Figure 7e shows the influence of viscosity in the form of PMMA concentration on strand length, width, spacing, and aspect ratio. Therefore, particles with a mean size of x_c_ = 16 nm were used for this purpose. Above a 5% PMMA, no strand formation is observed as it is already excessively viscous to form strands. In contrast, the larger particles still form strands even at 12.5% PMMA since the magnetic force is stronger (Figure S6). Interestingly, the strand length does not change between 1% and 5% PMMA, which can be explained by the non-linear dependence on PMMA concentration and viscosity. For lower PMMA concentrations, the length increases linearly again. The strand width and the spacing of the strands follow the same trend. From the experiments, it can be concluded that by varying the PMMA concentration, the strand length, width spacing, and resulting aspect ratio do not change much over a wide range (0.5–5 wt%).

In summary, the experimental results show a weak influence of the PMMA concentration on the strand length. At 7.5 wt%, PMMA strand formation abruptly stops. This is also confirmed by the simulations, where this concentration corresponds to an intrinsic viscosity of 0.8 mPa*s, a value at which strands are no longer formed according to the difference curve (Figure 7d). It should be noted that the PMMA concentration does not affect the viscosity linearly, as shown by Liu et al. [73]. For low PMMA concentrations, the flow and evaporation behavior of the solution plays an additional role. Due to the more pronounced movement of the liquid during evaporation, the movements of the liquid overlap with the inherent movements of the particles. This effect is not considered in the simulation.

### 3.3. Effect of the External Magnetic Field

**Figure 8:**
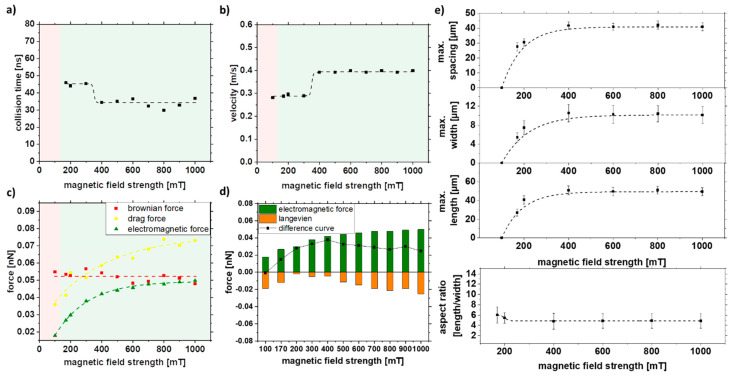
COMSOL simulation data of (**a**) collision time, (**b**) velocity, and (**c**,**d**) forces in dependence of the external magnetic field strength and experimental validation of (**e**) length, width, spacing, and aspect ratio of strands as a function of magnetic field strength in 5% PMMA solution.

In ferromagnetic materials, the relationship between field strength *H* and magnetization *M* is not linear and takes a hysteresis form. By increasing the field, one can increase the magnetization to high field magnetization (Figure S4). Therefore, the magnetic field strength was increased to observe its effect on strand formation. It can be seen that at low field strengths (<100 mT), strand formation no longer occurs. At the same time, it is evident that an increase in the field strengths to >~300 mT does not affect the strand size. Neither a faster collision nor an increased velocity could be observed after the simulation with increasing magnetic field strength (Figure 8a,b). As seen in Figure 3d–f and/or Figure S3, a saturation of magnetization occurs at ~300 mT. As a result, there is no increase in electromagnetic force and Langevin force with increasing magnetic field strength above approximately 300 mT (Figure 8c,d). An increase in the field strength would not lead to an increase in the magnetization of the particle and, thus, to longer strands. This could be confirmed experimentally, and only a maximum strand length of 55 nm and a width of 12 nm could be achieved for magnetic field strengths over 300 mT (Figure 8e).

The difference curve (Figure 8d) for a magnetic field strength of 100 mT leads to negative values indicating that strands are not formed. Above 300 mT, no change is visible due to reaching the saturation magnetization, which is also reflected in the experiments. With an increase of the magnetic field strength from 170 to 400 mT, a doubling of the force, from 0.014 to 0.038 nN, is observed in the difference curve, which correlates with the experimentally observed increase of the strand length from 25 to 50 µm. A way to change the magnetization or high field magnetization is possible with the change of the material system, which can influence the strand formation towards longer strands and is studied in the next chapter.

### 3.4. Effect of the Nanomaterial Composition

Since alloy targets may not be available in different mole fractions, a very convenient approach is synthesizing nanoparticles by ablating a pressed powder target [74,75]. Following this route, we have produced various iron-nickel alloy nanoparticles. Table 1 shows the mean particle size of the respective colloids. Since these were not centrifuged, these particles have a different distribution. As in the previous investigations, a particle size of 30 nm was selected for the simulation to compensate for magnetization’s sole effect. From the Slater–Pauling curve, it can be seen that the magnetic moment can be changed by the alloys. Fe, Fe_90_Ni_10_, Fe_63_Ni_36_, Fe_50_Ni_50_, Fe_20_Ni_80_, and Ni nanoparticles were produced for the series of experiments. Fe_63_Ni_36_, Fe_50_Ni_50_, and Fe_20_Ni_80_ were taken because they are industrially attractive and heavily used [76]. Additionally, Fe_90_Ni_10_ was taken here because it has one of the highest magnetizations of FeNi alloys in the literature [77]. Please note that the magnetic properties of the synthesized particles might change depending on whether the particles are synthesized from a bulk target (as is the case in the former sections) or from a pressed powder target as is in this section [78]. This difference is evident in the high field magnetization of Fe_50_Ni_50_, which is 34.5 Am²/kg in Table 2, but 52 Am²/kg in Figure 3. However, since we want to study the effect of the material composition in this section, this difference in magnetization is not relevant as long as the particles that are being compared were synthesized by the same (powder) route.

In Table 2, the specific values of the magnetometry measurements were extracted. The high field magnetization at 1 T *M*_hf_, the magnetization at 170 mT, the coercivity *H*_c_, and the remanent magnetization *M*_r_ were plotted. For the simulation, the crucial parameter is the magnetization at 170 mT because this is the strength of the magnets we use for the experiments. Here, as expected, it can be seen that the magnetization increases with increasing iron content, with Fe_90_Ni_10_ having an even higher magnetization, as can also be seen in the Slater–Pauling curve [79].

**Figure 9:**
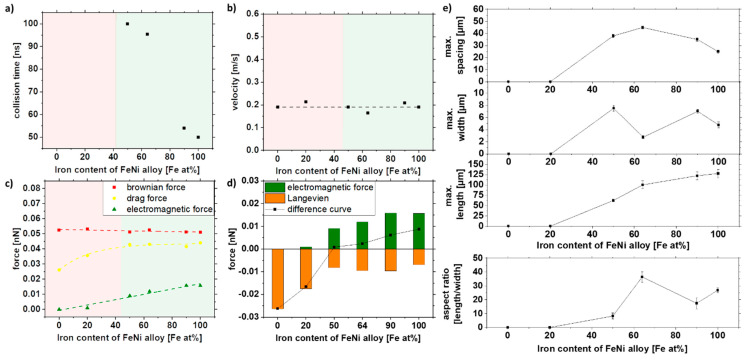
COMSOL simulation data of (**a**) collision time, (**b**) velocity, and (**c**,**d**) forces in dependence of the iron content of the FeNi alloy and experimental validation of (**e**) length, width, spacing, and aspect ratio of strands as a function of the iron content of the FeNi alloy in 5% PMMA solution.

Figure 9e shows the microscopy image analysis of the strands and the strand length of the 10% of the largest strands of the different alloys. It can be seen that with increasing iron content and associated magnetization of the particles, the strand length increases. Since the densities of iron and nickel are relatively close to each other at 7.87 and 8.91 g/cm³, hardly any difference in Brownian force is evident in our simulation. Thus, most of the change comes from the increase in magnetization alone. Nevertheless, as the electromagnetic force is weaker than the Brownian force, no change in velocity could be observed (Figure 9b,c). Since the attraction radius increases with magnetization, collisions occur more quickly. Fe_20_Ni_80_ and Ni, on the other hand, do not form strands, which may be due to the particle magnetization being too low, indicating that a minimum magnetization of 20 Am²/kg must prevail to form strands in these conditions. The simulations also show a good prediction of the strand formation. Ni and Fe_20_Ni_80_ do not have sufficient magnetization so that no collision occurs in the simulation (Figure 9a). It could be shown experimentally and via simulation that the particles need a minimum magnetization to form strands, and this is around 20.8 Am^2^/kg for our system.

The width and spacing of the strands, on the other hand, show a different trend. A maximum in width and spacing can be seen at 50–60 at% and decreases with higher iron content. Fe_64_Ni_36_ shows a smaller width than the other samples, so the aspect ratio is significantly higher than the other compositions. This may be because the samples have slightly different size distributions, resulting in slight deviations in strand formation (Table 2). As shown in Figure 6e, size distribution significantly affects strand formation, as larger particles result in longer and broader strands.

As shown in Figure 9e, the strand length does not increase significantly with increasing iron content after a value of 64% Fe (invar). When looking at Table 2, it can be seen that there is hardly any difference in the magnetization at 170 mT for the iron-rich alloys, so that we conclude that higher iron contents do not necessarily lead to longer strands and that the magnetization has to be strongly increased, to increase the strand length significantly. With the bimodal size distribution of Fe_50_Ni_50_ nanoparticles, the size-dependent magnetic properties in the composite can be investigated. Therefore, the size-separated Fe_50_Ni_50_ nanoparticles from the bulk target were used for further investigation.

Summarizing the results of this section, simulation and experiments predict no strand formation below 50 at% Fe and a linear increase in strand length with increasing iron content. Thereby, both simulation and experimental data show a similar slope of 1.56 × 10^−4^ nN/(at% Fe) and 1.45 µm/(at% Fe), respectively.

### 3.5. Magnetic Properties of the Composite

**Figure 10:**
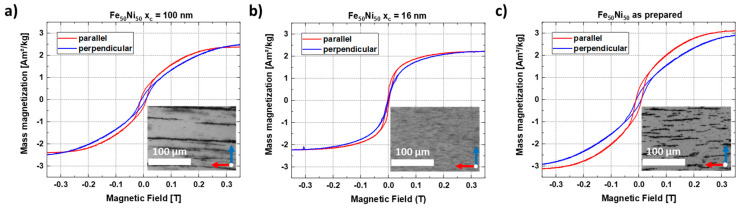
In-plane hysteresis loops measured parallel and perpendicular to the FeNi nanostrands of the 0.2 wt% composite at T = 300 K using colloids with mean sizes of (**a**) x_c_ = 100 nm, (**b**) x_c_ = 16 nm, and the (**c**) as-prepared colloid.

The magnetic properties of the FeNi-PMMA composites with strands of different particle sizes were investigated. For this purpose, the composite materials were lifted from the glass substrate using adhesive tape, which has a very low weight, lower than the original substrate and a very small, diamagnetic, and thus linear background signal. *M*(*H*) curves were recorded up to fields of 9 T at room temperature, with one measurement being performed for parallel and one for perpendicular orientation of the strands relative to the external magnetic field (Figure 10). While the trends in the high field regions appear mostly identical, except for minor geometry effects due to imperfect sample shapes, the low field region gives clear indications regarding the orientation of the samples. We can consistently observe a faster increase of magnetization of the parallel compared to the perpendicular strand orientation for the x_c_ = 100 nm particles (Figure 10a), clearly showing the macroscopic properties resulting from the microscopic local orientation and alignment of the particles’ easy magnetic axes within the strands. Meanwhile, the smaller x_c_ = 16 nm particles (Figure 10b) display small strand formation with small spacing between the strands (inset), and as a result, it displays a difference in saturation behavior. The as-produced sample (Figure 10c) also shows clear strand formation and fast saturation in parallel orientation. Therefore, it is shown that there is a strong particle size dependence for both the formation of nanostrands and the resulting anisotropic magnetic behavior. To crosscheck this trend, a reference sample without formed nanostrands (Figure S8) was analyzed, confirming the lack of preferred orientation. For this sample, no apparent differences were visible in the low field region, revealing the effectiveness of the utilized growth process and magnetic field-induced alignment of the nanostrands.

In the last step, the Fe_50_Ni_50_ nanoparticles were also evaluated for electrical conductivity, which is relevant for applications in conductive transparent polymers, such as rear windows in automobiles. Therefore, the colloid was dropped onto an interdigital electrode. Considering the settings resistance series (shunt), the voltage drop across this shunt gives the current. The current at the electrode without nanostrands is 0.43 µA, while the current of the electrode with the formed strands is 0.43 mA, which is almost a factor of 1000 higher (Figure S9a). Moreover, in long-time measurements of more than 1 h, no conductivity change was observed, indicating good stability of the particles and strands (Figure S9b). Accordingly, the nanoparticles in the strands are densely packed with good particle connectivity, thereby increasing the electrical conductivity.

## 4. Conclusions

Laser synthesized magnetic FeNi nanoparticles were successfully size-separated and used for strand formation in a polymer matrix. The bimodal size distribution of the laser-ablated Fe_50_Ni_50_ particles was the optimal material to investigate the size-dependent studies of strand formation and magnetic properties in the composite. All size-separated colloids have a monomodal size distribution with a PDI smaller than 0.3 and have an average size of 7.5 ± 2.4, 15.7 ± 3.1, and 98.5 ± 28.1 nm, respectively. Simulations with the finite-element software COMSOL Multiphysics have been established as a variable and straightforward method to predict strand formation using Brownian, drag, and electromagnetic force. The influence of particle size, viscosity, external magnetic field strength, and composition of the particles could be simulated and experimentally confirmed. Compared to the nanoparticles obtained from the powder target, the Fe_50_Ni_50_ nanoparticles from the bulk target form much longer strands with higher aspect ratios. Therefore, these nanoparticles were used for the investigations. In the course of the experimental study, the optimal parameters for Fe_50_Ni_50_ were found. It could be shown that small particles do not form strands due to superparamagnetic behavior; a particle size larger than 20 nm is needed to realize strand formation. With increasing particle size, the strand length also increases with x². Higher viscosities in the nanoparticle-polymer solution negatively influence the strand formation due to the increased Brownian force. At a viscosity of 0.8 mPa*s, no collision of the particles could be observed in the simulation. Since the hysteresis curve reaches its saturation at 300 mT, no change in strand length is evident by increasing the magnetic field strength. Hence, our results show that our COMSOL simulation can predict the strand formation and determine optimal parameters independent of the material system. It was also demonstrated that the strand length does not increase significantly with increasing iron content from about 64% Fe (the invar composition) because the magnetization at 170 mT is hardly changed. For possible applications, such as modular microswimmers or transparent conductive composites, we showed the control of the magnetic anisotropy and the electrical conductivity, increasing by 1000 when nanostrands are formed.

## Figures and Tables

**Table 1:** Centrifugation protocol for particle size x < 10 nm, 10 nm > x > 50 nm, and x > 50 nm.

Size	Step	Volume (mL)	RPM	Time (min)	Description
x < 10 nm	i	50	2000	36	use supernatant for second step
	ii	2	18,000	20	collect supernatant
10 nm > x > 50 nm	i	50	2000	36	use supernatant for second step
	ii	2	18,000	20	collect pellet
x > 50 nm	i	50	4000	9	centrifuged 7 times and collect pellet

**Table 2:** Density and magnetic properties of different iron-nickel alloys extracted from the hysteresis curve in Figure S7.

Material	ρ (g/cm³)	Mean Size (nm)	M_1T_ (Am^2^/kg)	M_170mT_ (Am^2^/kg)	H_c_ (mT)	M_r_ (Am^2^/kg)
Ni	8.91	13.6 ± 9.4	1.1	0.17	−0.6	0.001
Fe_20_Ni_80_	8.70	17.8 ± 13.4	11.7	6.8	−0.7	0.03
Fe_50_Ni_50_	8.40	13.1 ± 9.0	34.5	20.8	−0.9	0.6
Fe_64_Ni_36_	8.25	11.9 ± 5.7	46.1	24.7	−3.3	1.6
Fe_90_Ni_10_	7.98	14.5 ± 7.7	54.7	28.3	−6.0	2.3
Fe	7.87	16.5 ± 8.5	50.5	24.9	−7.1	2.3

## Supplementary Materials

The following are available online at https://www.mdpi.com/article/10.3390/nano11082095/s1, Figure S1: Mean inter-particle distance for the COMSOL simulation, Figure S2: relative mass frequency of Fe_50_Ni_50_ nanoparticles, synthesised by a ps-laser, Figure S3: EDX line-scan and mapping of the synthesized Fe_50_Ni_50_ nanoparticle. Figure S4: M(H) curve, Temperature-dependent field cooled (FC) and zero field cooled (ZFC) magnetization curves of Fe_50_Ni_50_ nanoparticle as synthesized, Figure S5: SEM images of Fe_50_Ni_50_ particle with a mean size of x_c_ = 8 nm dried under a magnetic field of 170 mT, Figure S6: Comparison of Fe_50_Ni_50_ strand lengths formed in a PMMA-acetone solution with variable PMMA amount for particles a) 10 nm < x < 50 nm (x_c_ = 15.7 nm) and b) x > 50 nm (x_c_ = 98.5 nm), Figure S7: M(H) curve of different FeNi alloy nanoparticle as synthesized at 300 K, Figure S8: In-plane hysteresis loops measured parallel and perpendicular to the FeNi particles of the 0.2 wt% composite at T = 300 K without strand formation, Figure S9: Conductivity measurement of the Fe_50_Ni_50_ particles without and with formed FeNi nanostrands.
