# Supplementary material for: Formation of Fe-Ni Nanoparticle Strands in Macroscopic Polymer Composites: Experiment and Simulation
Source: Nanomaterials (Basel). 2021 Aug 18;11(8):2095. doi: 10.3390/nano11082095 (PMC8398175; doi:10.3390/nano11082095)
Supplement: Supplementary file 1 [file nanomaterials-11-02095-s001.zip › nanomaterials-1318484-supplementary.pdf]

nanomaterials

Supplementary Materials

Formation of Fe-Ni Nanoparticle Strands in Macroscopic Polymer Composites: Experiment and Simulation

Ruksan Nadarajah [1], Leyla Tasdemir [1], Christian Thiel [2], Soma Salamon [3], Anna S. Semisalova [3], Heiko Wende [3], Michael Farle [3], Stephan Barcikowski [1], Daniel Erni [2] and Bilal Gökce [1,4,*]

1 Technical Chemistry I, Center for Nanointegration Duisburg-Essen (CENIDE), University of Duisburg-Essen, Universitaetsstr. 7, 45141 Essen, Germany; ruksan.nadarajah@uni-due.de (R.N.); cakir_leyla@hotmail.com (L.T.); stephan.barcikowski@uni-due.de (S.B.)
2 General and Theoretical Electrical Engineering (ATE), Center for Nanointegration Duisburg-Essen (CENIDE), University Duisburg-Essen, 47048 Duisburg, Germany; christian.thiel.ate@uni-due.de (C.T.); daniel.erni@uni-due.de (D.E.)
3 Faculty of Physics, Center for Nanointegration Duisburg-Essen (CENIDE), University of Duisburg-Essen, Lotharstr. 1, 47057 Duisburg, Germany; soma.salamon@uni-due.de (S.S.); anna.semisalova@uni-due.de (A.S.S.); heiko.wende@uni-due.de (H.W.); michael.farle@uni-due.de (M.F.)
4 Materials Science and Additive Manufacturing, University of Wuppertal, Gaußstr. 20, 42119 Wuppertal, Germany
* Correspondence: goekce@uni-wuppertal.de

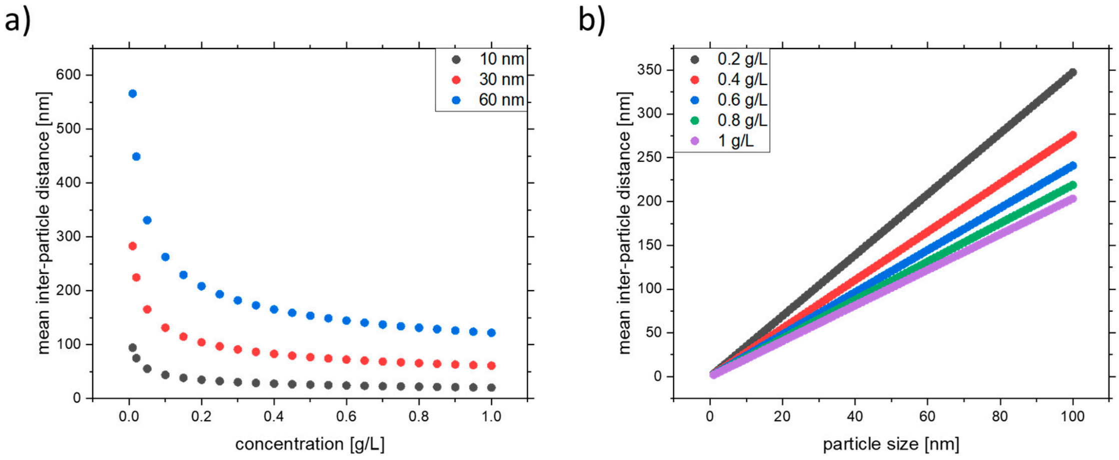

Figure S1. Mean inter-particle distance in dependence of a) concentration and b) particle size for the COMSOL simulation.

The concentration of the solution was controlled by the inter-particle distance. Here, the inter-particle distance was approximated by the Wigner-Seitz radius:

$$< r > \sim (\frac{3}{4\pi n})^{\frac{1}{3}}$$

Where n indicates the particle density $n = \frac{N}{V}$. Figure S1 shows the dependence of particle concentration and size on inter-particle spacing. It can be seen that small particle size and low concentration, leads to higher inter-particle spacings. The inter-particle distance increases linearly with particle size to $y = x$ and as the concentration increases it decreases with the function $y = \frac{1}{x}$.

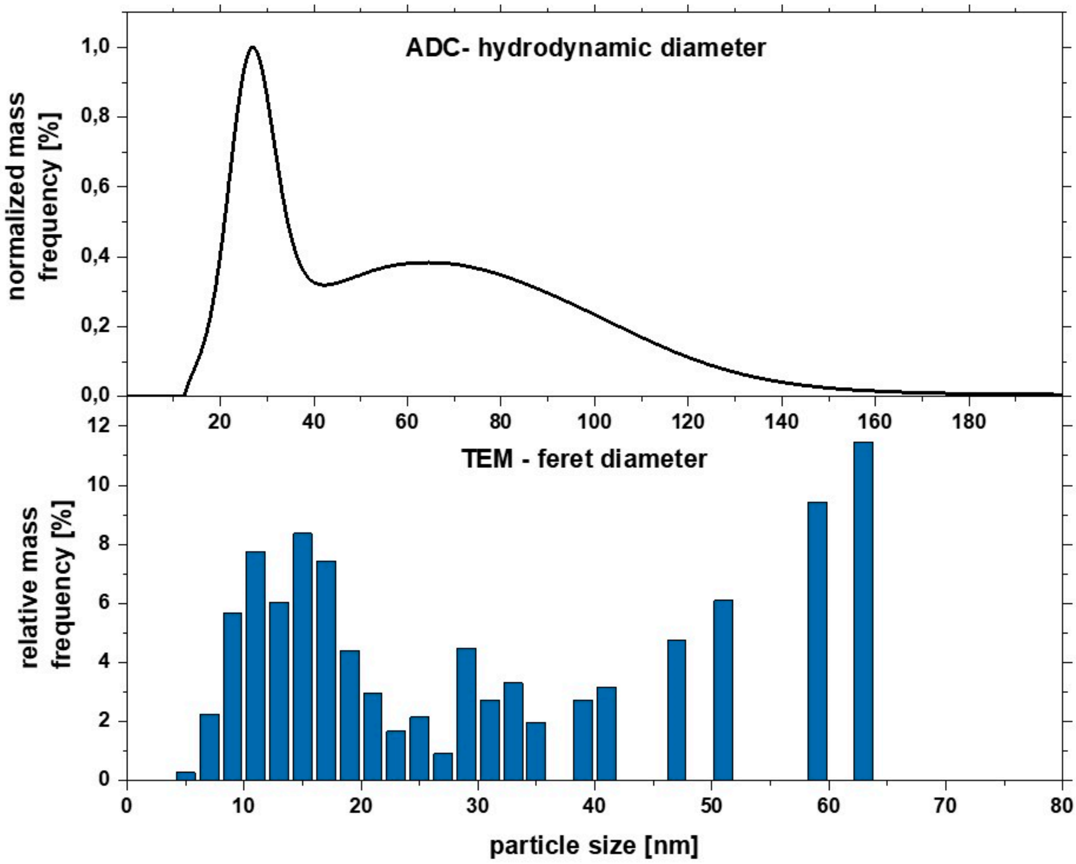

Figure S2: relative mass frequency of $Fe_{50}Ni_{50}$ nanoparticles, synthesised by a ps-laser (10 ps, 100 kHz, 10 J/cm²).

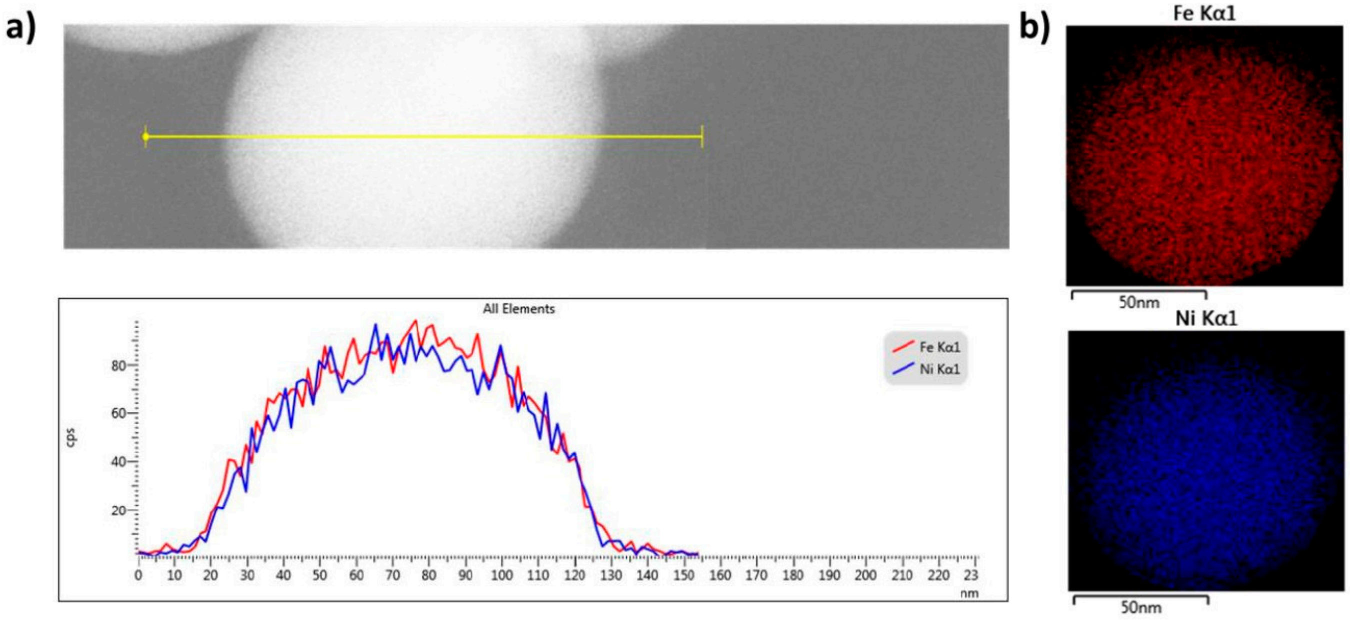

Figure S3. EDX **a**) line-scan and **b**) mapping of the synthesized $Fe_{50}Ni_{50}$ nanoparticle.

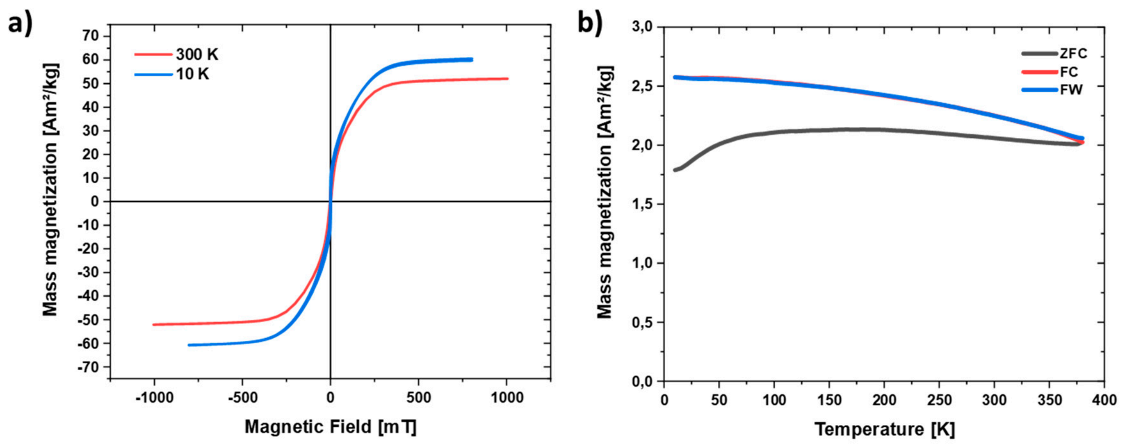

Figure S4. (**a**) M(H) curve of $Fe_{50}Ni_{50}$ nanoparticle as synthesized at 300 K (red) and 10 K (blue). (**b**) Temperature-dependent field cooled (FC, red) and zero field cooled (ZFC, black) magnetization curves measured in 2 mT field for the respective FeNi nanoparticles.

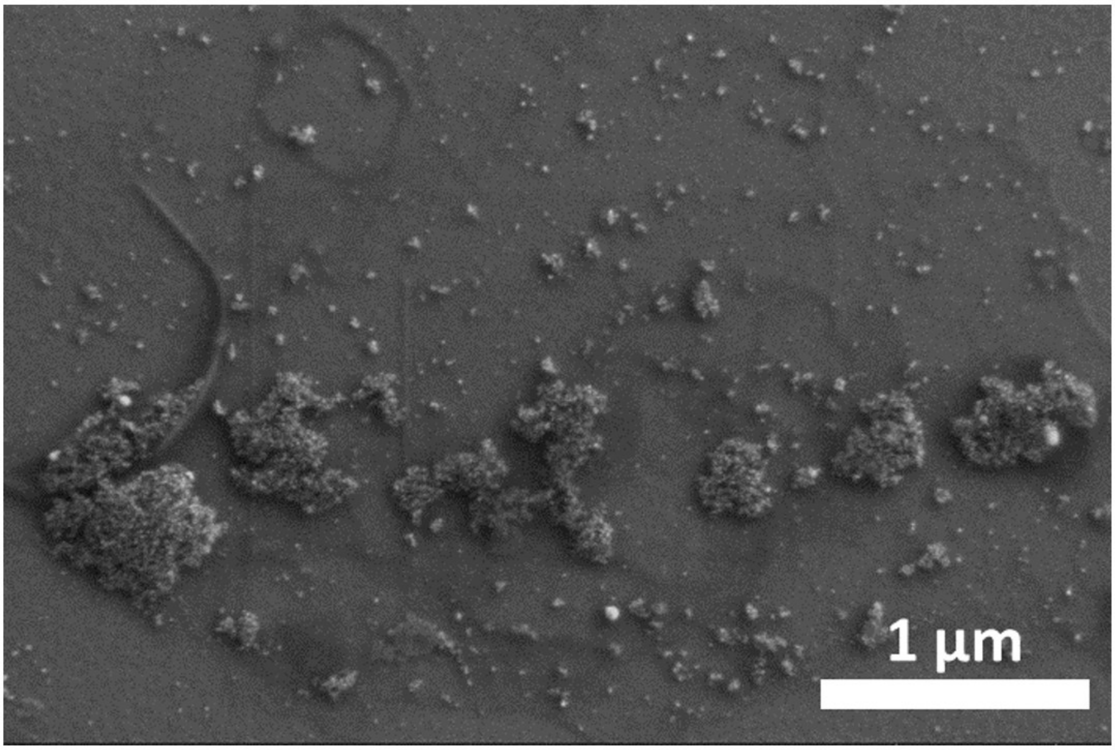

Figure S5. SEM images of $Fe_{50}Ni_{50}$ particle with a mean size of x_c = 8 nm dried under a magnetic field of 170 mT.

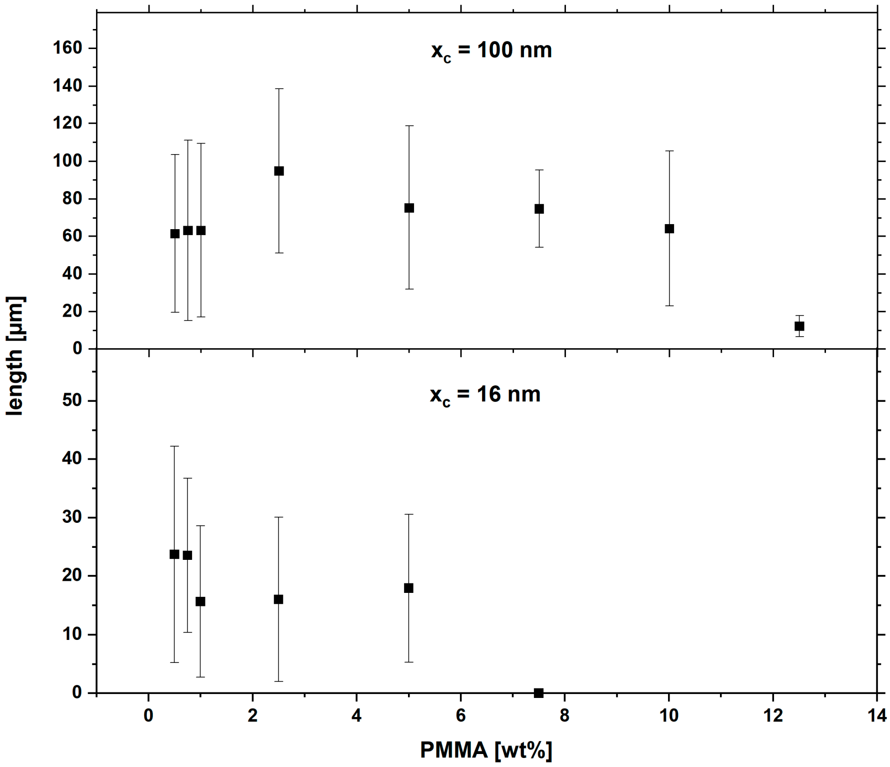

Figure S6. Comparison of $Fe_{50}Ni_{50}$ strand lengths formed in a PMMA-acetone solution with variable PMMA amount for particles **a**) 10 nm < x < 50 nm (x_c = 15.7 nm) and **b**) x > 50 nm (x_c = 98.5 nm).

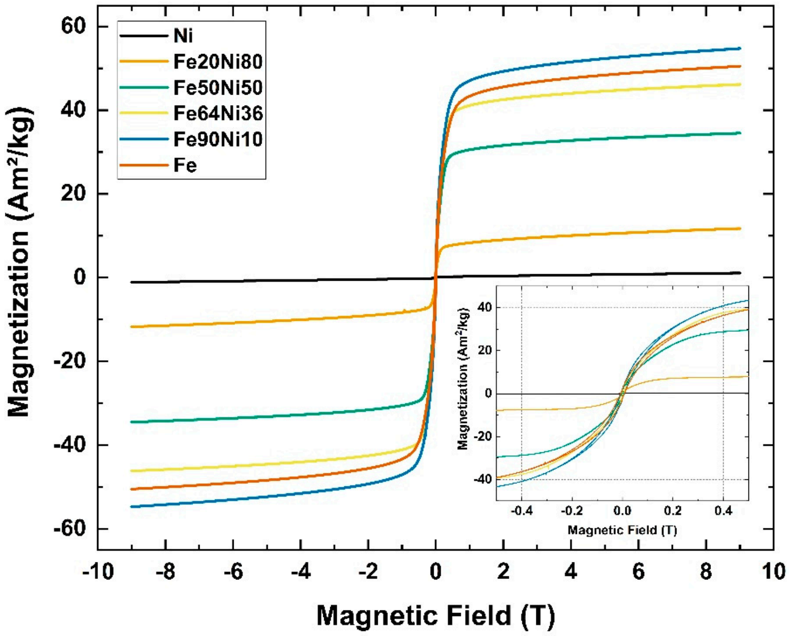

Figure S7. M(H) curve of different FeNi alloy nanoparticle as synthesized at 300 K.

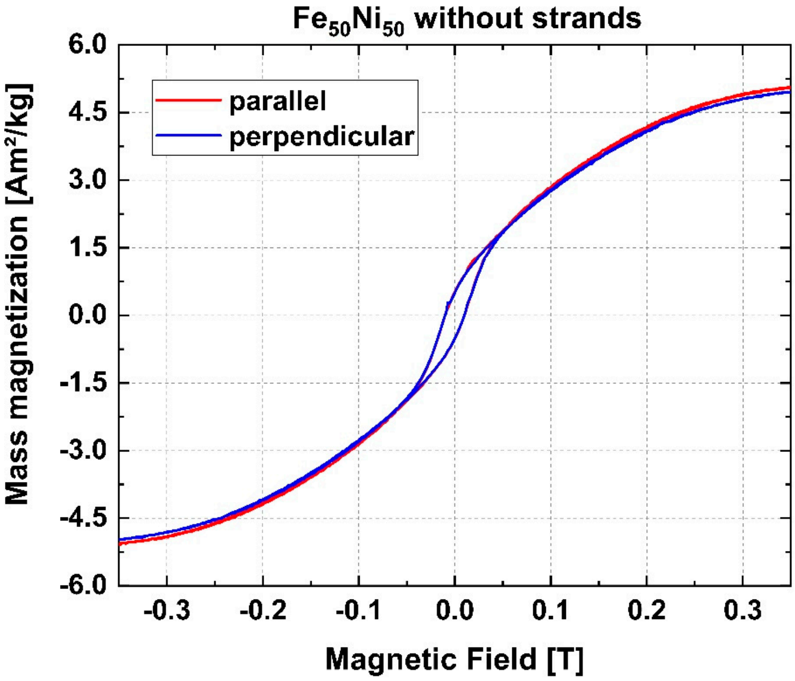

Figure S8. In-plane hysteresis loops measured parallel and perpendicular to the FeNi particles of the 0.2 wt% composite at T = 300 K without strand formation.

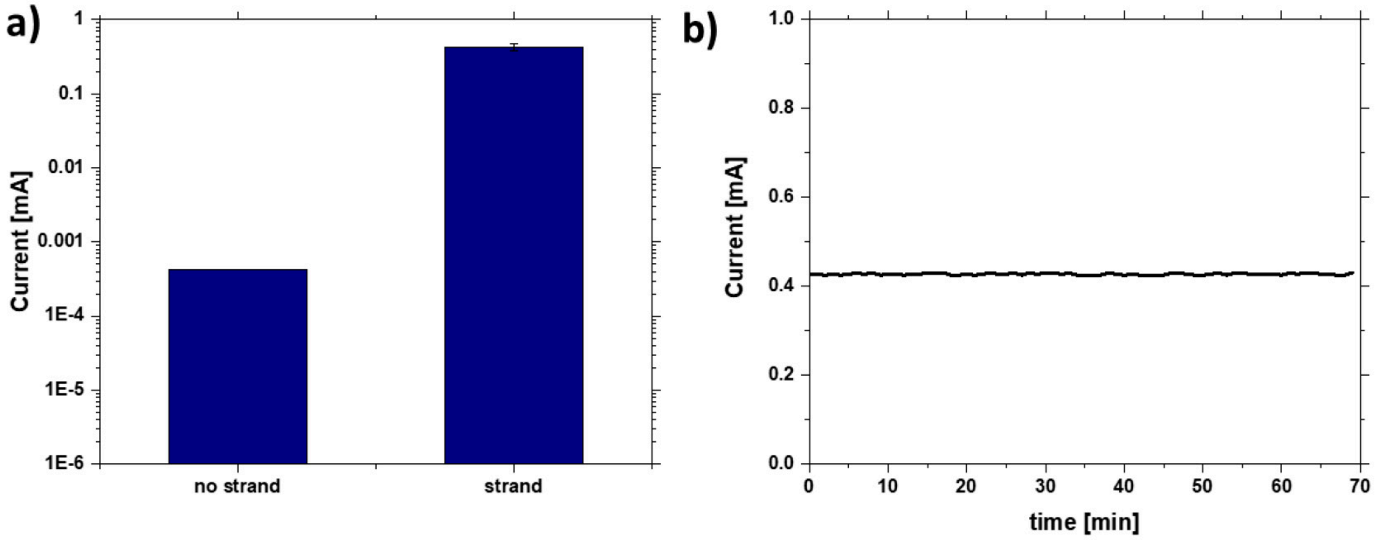

Figure S9. a) Conductivity measurement of the $Fe_{50}Ni_{50}$ particles without and with formed FeNi nanostrands and b) long-time conductivity test.
